# Treatment of gingival defects with gingival mesenchymal stem cells derived from human fetal gingival tissue in a rat model

**DOI:** 10.1186/s13287-017-0751-7

**Published:** 2018-02-05

**Authors:** Jing Li, Shi-qing Xu, Kai Zhang, Wen-jian Zhang, Hong-lin Liu, Zhen Xu, Hong Li, Jin-ning Lou, Li-hong Ge, Bao-hua Xu

**Affiliations:** 1 Dental Medical Center, China–Japan Friendship Hospital, Beijing, 100029 People’s Republic of China; 2 Institute of Clinical Medical Sciences, China–Japan Friendship Hospital, Beijing, 100029 People’s Republic of China; 3 Department of Pediatric Dentistry, Peking University School and Hospital of Stomatology, Beijing, 100081 People’s Republic of China

**Keywords:** Gingival defect, Gingival mesenchymal stem cells, Fetal stem cells, Transplantation

## Abstract

**Background:**

The study aimed to evaluate the efficacy and safety of gingival mesenchymal stem cells (GMSCs) from human fetal gingival tissue used for treating gingival defects in a rat model.

**Methods:**

GMSCs were isolated from human fetal gingival tissue and identified by flow cytometry for nestin, Oct4, vimentin, NANOG, CD105, and CD90. The immunogenicity of GMSCs was analyzed by mixed lymphocyte reactions; the tumorigenicity of GMSCs was evaluated by xenotransplanting into nude mice. The gingival defect animal model was established by mechanical resection in rats. GMSCs were transplanted into the defective area, and the regeneration of gingival tissue was observed twice weekly. Four weeks after transplantation, the gingival tissue was surgically cut down, and the graft was analyzed by immunohistochemistry staining for human mitochondrial antigens and rat CD3 and CD20.

**Results:**

GMSCs from human fetal gingival tissue positively expressed nestin, Oct4, vimentin, NANOG, CD105, and CD90. There was no cell aggregation after mixed lymphocyte reactions, and interleukin-2 did not increase. Inoculation of GMSCs into nude mice for 6 months showed no tumor formation. GMSCs were transplanted into the gingiva defects of rats. One week after transplantation, the defect area was reduced, and after 3 weeks the morphology and color of local gingival tissue was similar to normal gingival tissue, and gingival height was the same as the normal control group.

**Conclusions:**

Using GMSCs from human fetal gingival tissue to treat gingival defects is a safe and effective innovative treatment method.

## Background

Periodontitis is an inflammatory and destructive disease that occurs in the gingiva, periodontal ligaments, alveolar bone, and cementum, and is one of the oldest and most common oral diseases. The incidence of periodontal disease in different countries varies from 50 to 90% [1, 2] and is the main cause of tooth loss in adults. The typical feature of the stationary phase of periodontitis is defects of the gingiva and other periodontal tissues. The incidence of gingival recession increases with age, with 58% of people over age 30 having at least 1 mm of gingival recession and nearly all people over age 50 having varying degrees of gingival recession [2]. In some elderly patients with moderate or severe periodontitis, a wide range of severe gingival defects and alveolar bone loss exists. Severe gingival tissue defects not only affect patients’ appearance, but also their pronunciation and eating.

Basic periodontal treatment can only remove plaque and calculus to control inflammation, while the final goal of periodontal treatment is to repair and reconstruct the periodontal tissue. At present, the clinical symptoms caused by root exposure often require surgical methods, such as the laterally positioned flap, the free gingival graft, and guide tissue regeneration, to cover the exposed root surface and improve clinical symptoms. Surgical treatment often results in trauma, covers a limited treatment area, requires adequate healing time, and is unaffordable to many, and long-term chronic inflammation can lead to tissue sensitivity. Although several treatment methods exist to regenerate periodontal tissue, histological analysis shows that most can only form fibrous connective tissues and do not achieve true periodontal regeneration.

Stem cell technology provides new methods for periodontal regeneration. Stem cells can proliferate in vitro, and after transplantation can differentiate into desired tissues to repair defects. An important issue is to separate high-quality seed cells from available sources for tissue regeneration.

Fetal stem cells can be isolated from fetal tissue and, being more primitive than adult stem cells, can maintain self-renewal and differentiation potential. Under certain conditions they can differentiate into various tissues. There are at present no studies transplanting cultured GMSCs from human fetal gingival tissue into animals with gingival defects to repair periodontal tissue. We therefore cultured GMSCs from human fetal gingival tissue in vitro, transplanted the cells into the gingival defects of a rat model, and evaluated their therapeutic efficacy and safety.

## Methods

### Isolation and culture of human fetal GMSCs

Gingival tissues at the 10th–12th gestational week were obtained from the maxillary and mandibular alveolar ridge under sterile conditions. The gingival tissues were cut into small pieces, digested with 2 ml of 0.2% type I collagenase (Sigma, USA) in a 37 °C water bath for 30 min, and washed twice with DMEM/F12 (Sigma) followed by centrifugation. Cells were cultured in DMEM/F12 containing 10% fetal calf serum for stem cells (Biochrom, Germany), 40 μg/L leukemia inhibitor factor (LIF; PeproTech, USA), 10 μg/L basic fibroblast growth factor (bFGF; PeproTech), and 10 μg/L epidermal growth factor (EGF; PeproTech) in 25-cm^2^ T-flasks at 37 °C in 5% CO_2_. The medium was changed every 3 days. Upon reaching 80% confluence, adherent cells were detached with 0.1% trypsin/0.1% ethylenediaminetetraacetic acid (EDTA), passaged at 1:3 ratios, and cultured for further study. The cells used in this study were at passage 3–5.

### Identification of human fetal GMSCs

#### Flow cytometric analysis

Cells in the logarithmic growth phase were digested with 0.1% trypsin/0.1% EDTA into single cell suspensions. Cells were respectively labeled with rabbit anti-human nestin (Millipore, Germany), Oct4 (Cell Signaling, USA), vimentin (Abcam, UK), NANOG (Abcam), and CD90 (Abcam) antibodies, and mouse anti-human CD105 (Abcam) antibody. About 1 × 10^6^ cells were used for detection of each molecule. Alexa Fluor 488-conjugated anti-mouse and anti-rabbit were used as secondary antibodies. Labeled cells were thoroughly washed followed by centrifugation three times, and then resuspended in PBS solution. Flow cytometry (EPICS XL; Beckman, USA) was used to detect fluorescence intensity and the number of positive cells.

#### Immunohistochemical staining

Human fetal gingival tissue specimens were fixed with 4% polyformaldehyde solution, paraffin embedded, and cut into 5-μm slices. Sections were stained for nestin, Oct4, vimentin, NANOG, CD105, and CD90 according to standard techniques, observed under a microscope (OLYMPUS BX53; OLYMPUS, Japan), and photographed.

### Animal experiments

#### Gingival defects animal model

Six-week-old male Wistar rats and 8-week-old Nu/Nu male mice were purchased from Beijing Vital River Laboratory Animal Technology Company (quality certificate: SCXK (Beijing) 2012-0001). All animals were housed in the specific pathogen free (SPF) facility (quality certificate: SYXK (Beijing) 2010-0011) in the Institute of Clinical Medical Sciences of the China–Japan Friendship Hospital, and rats were maintained on a 12-h light:12-h dark cycle with free access to rodent chow and water.

Twenty-four Wistar rats were randomly divided into four groups: control group, gingival defect model group, transplanted GMSC group, and saline group. Gingival defects were established in the latter three groups. Mandibular incisor labial gingival tissue was resected by an electrosurgical generator, and the wound dressed with a surgical blade. The diameters of gingival defects were over 5 mm, deep to hard tissue.

#### Transplantation of human fetal GMSCs

Stem cells were transplanted 6 days after the resection of the mandibular incisor labial gingival tissue. Human fetal GMSCs between passages 3 and 5 were harvested by digestion, washed twice, and then suspended in DMEM/F12 at a density 2 × 10^6^ cells/100 μl. A 1-ml sterile syringe with a disposable scalp needle was used to aspirate the cell suspension, and a hand centrifuge was used to gather the cells at the head of the needle in a mass about 5 mm long. After centrifugation, the GMSC cell mass (2 × 10^6^ cells/rat, about 10–15 μl) was injected around the gingival defect. The saline group was injected with the same volume of saline.

Four weeks after transplantation, the gingival tissues where stem cells were transplanted and normal gingival tissue at the same location were removed, fixed with 4% polyformaldehyde solution, paraffin embedded, and cut into 5-μm slices. Sections were stained with mouse anti-human mitochondrial (Mito) antibody (Millipore), rabbit anti-rat CD3 antibody (Abcam), and goat anti-rat CD20 antibody (Santa Cruz, USA) according to standard techniques. The results were observed under a microscope (OLYMPUS BX53) and photographed.

#### Tumorigenicity assay

Human fetal GMSCs were suspended in DMEM/F12 medium at a density of 2 × 10^6^ cells/100 μl. Six nude mice were anaesthetized and 2 × 10^6^ GMSCs were subcutaneously inoculated into the backs of each nude mouse. The grafts were observed every 3 days and photographed.

### Immunogenicity assay of human fetal GMSCs

Human peripheral blood mononuclear cells (PBMC) were isolated from healthy donors by human peripheral blood lymphocyte separation solution (Tian Jin Hao Yang Biological Manufacture Co., Ltd, China) density gradient centrifugation, cultured in RPMI-1640 medium (Gibco, USA) supplemented with 10% FBS, 2 mM glutamine, 100 U/ml penicillin, and 100 mg/ml streptomycin (North China Pharmaceutical Limited by Share Ltd, China), and incubated at 37 °C, 5% CO_2_. Lysates of human fetal GMSCs were prepared by an ultrasonic cell disrupter and adjusted to a concentration of 250 μg/ml. PBMCs were divided into three groups: control group, 2 × 10^6^ PBMCs/well were cultured in 24-well plates in normal medium as already described; PHA + PMA group, 10 μg/ml PHA + 0.1 μg/ml PMA with 2 × 10^6^ PBMCs; and stem cell group, 250 μg/ml GMSC lysate with 2 × 10^6^ PBMCs. Each group was cocultured for 24 and 48 h, the culture medium was collected, and the concentration of IL-2 was measured by ELISA (Human IL-2 ELISA Kit; Beijing 4A Biotech Co., Ltd, China).

### Statistical analysis

Analyses were conducted with SPSS 19.0 software and the data were expressed as means ± SD. The *t* test was used for comparison between two groups and one-way analysis of variance (ANOVA) for comparison among multiple groups. *P* < 0.05 and *P* < 0.01 denoted statistical significance.

## Results

### Culture and identification of human fetal GMSCs

**Fig. 1:**
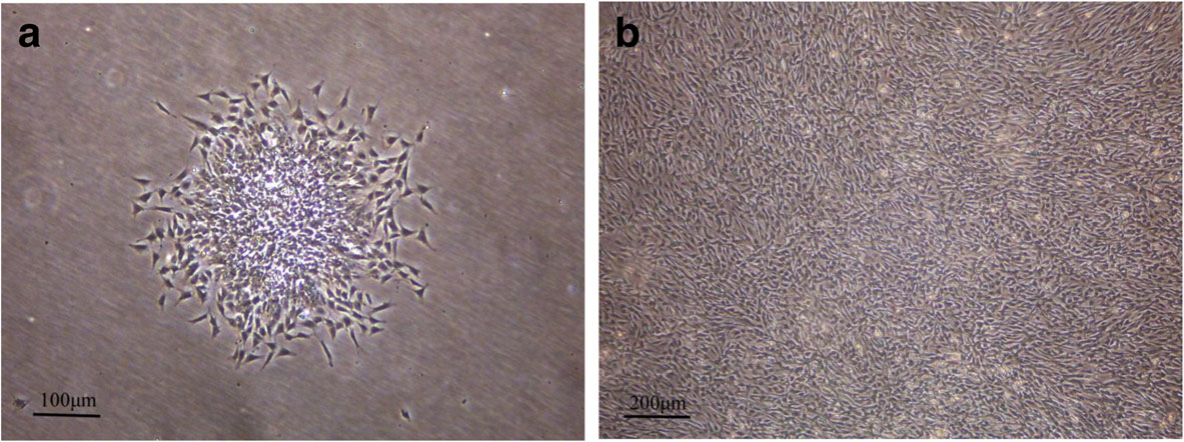
Culture of GMSCs. **a** Cell clone of primary human fetal GMSCs. **b** Primary human fetal GMSCs cultured for 14 days

Human fetal GMSCs adhered to the bottom of flasks after overnight culture. These cells proliferated rapidly and formed clones 3 days later (Fig. 1a). After culturing for about 14 days, the cells exhibited polygonal or fusiform morphology with characteristics of monolayer growth and contact inhibition (Fig. 1b).

### Identification of human fetal GMSCs

**Fig. 2:**
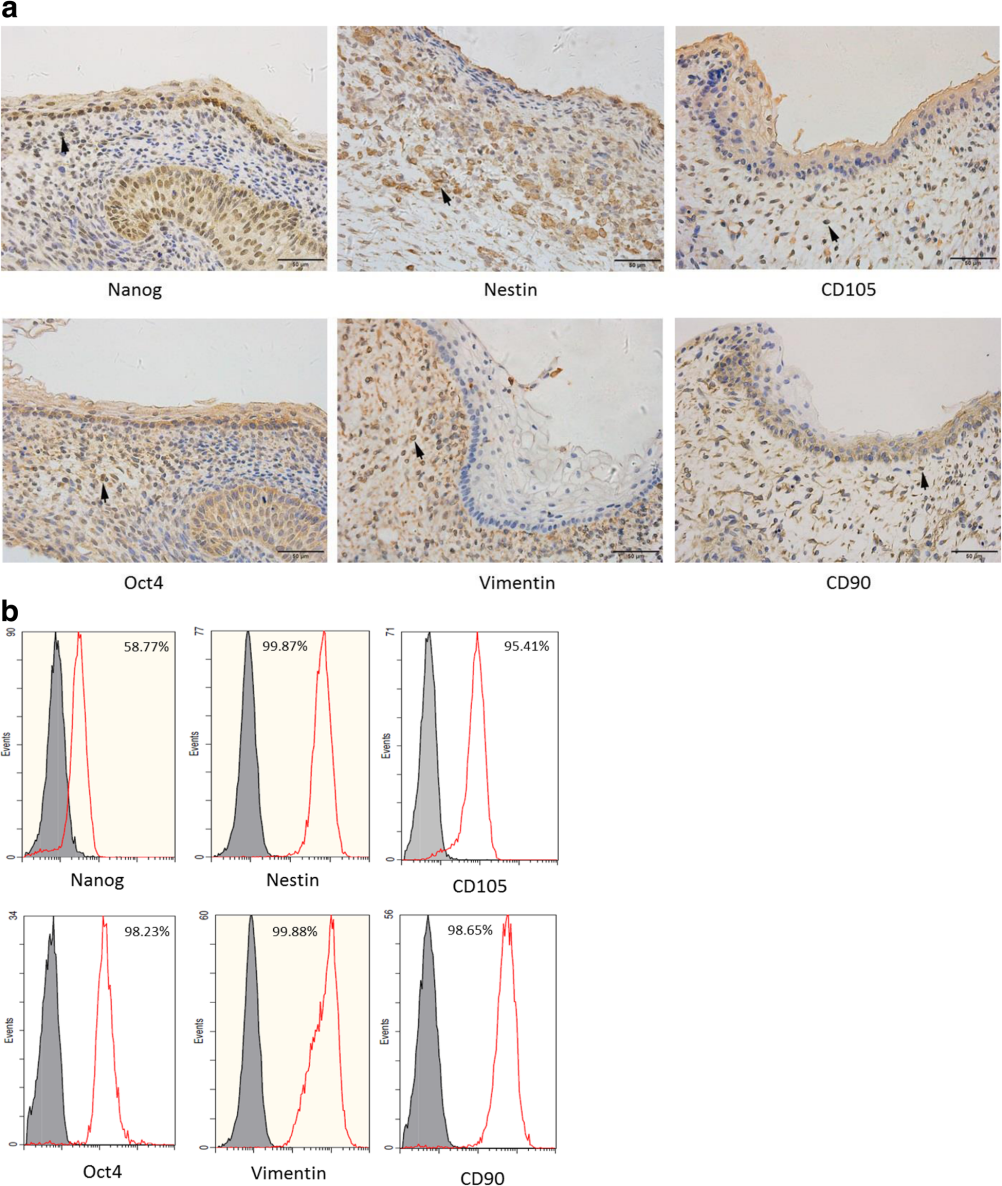
Identification of human fetal GMSCs. **a** Immunohistochemical staining of nestin, Oct4, vimentin, NANOG, CD105, and CD90 in human fetal gingival tissue (arrows denote positive cells). **b** Human fetal GMSCs stained for nestin, Oct4, vimentin, NANOG, CD105, and CD90 and analyzed by flow cytometry, revealing positive expression of all markers

In human fetal gingival tissue, nestin, Oct4, vimentin, NANOG, CD105, and CD90 were found by immunohistochemical staining (Fig. 2a). Therefore, these markers were used for identification of GMSCs. By flow cytometry, cultured human fetal GMSCs were strongly positive for nestin, Oct4, vimentin, NANOG, CD105, and CD90 (Fig. 2b). These results confirmed that the cells isolated from human fetal gingival tissues were mostly mesenchymal stem cells.

### Transplanting of human fetal GMSCs

**Fig. 3:**
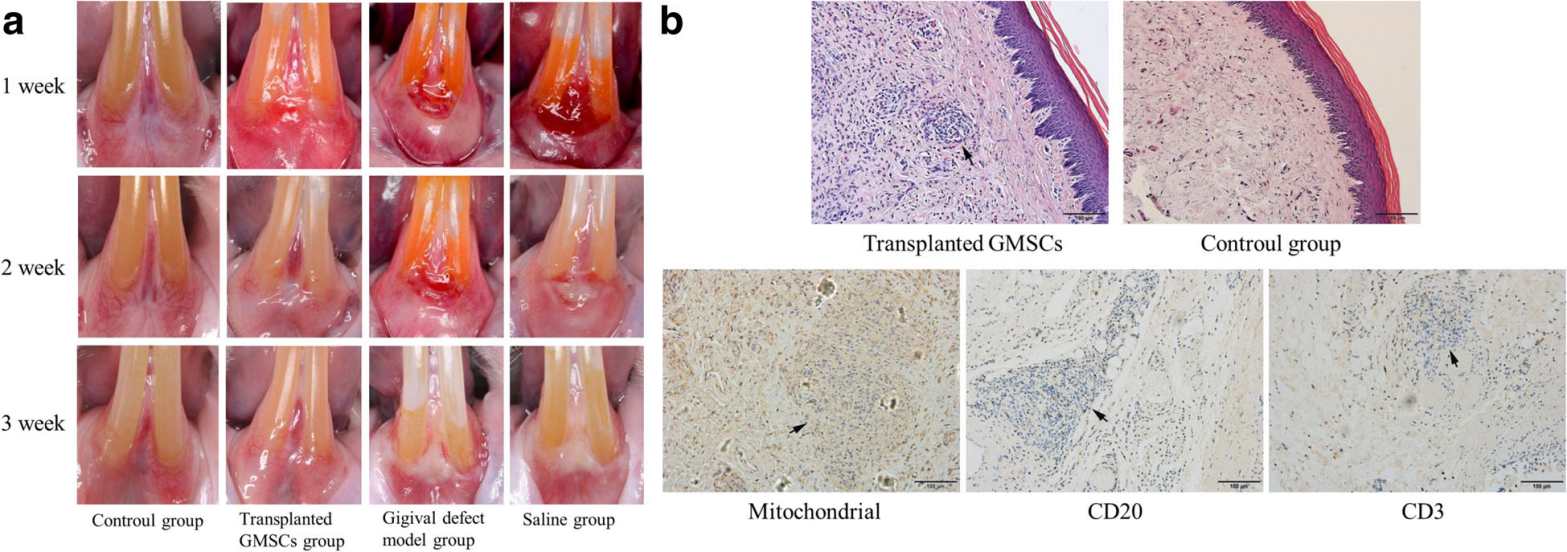
Preparing the gingival defect animal model and transplanting human fetal GMSCs. **a** Six days after gingival tissue was resected, human fetal GMSCs were transplanted to the labial gingiva defect of the anterior teeth in rats. In the control group, rats were not intervened with during the experimental period. In the transplanted GMSC group, after the gingival defect model was established, 2 × 10^6^ human fetal GMSCs were transplanted into the defect area of each rat; 3 weeks after stem cell transplantation, gingival tissues were almost the same as the control group. In the gingival defect model group, after resection of gingival tissue, no other procedures were performed; 3 weeks after resection of gingival tissue, the defect area was not healed completely. In the saline group, saline was used instead of stem cells in the defect area, and gingival healing was similar to that in the third group. **b** Four weeks after transplantation, gingival tissues where stem cells were transplanted and normal tissue was removed; histological sections stained with HE, mouse anti-human mitochondrial (Mito) antibody, rabbit anti-rat CD3 antibody, and goat anti-rat CD20 antibody. Mito section displays negative result, CD3 and CD20 sections display negative results; arrows denote transplanted stem cell masses. GMSC gingival mesenchymal stem cell

The rat gingival defect animal model was established by surgical technique. Six days later, human fetal GMSCs were transplanted to the labial gingiva defect of the anterior teeth. One week after transplantation, the defect area was reduced in the cell transplantation group compared with that of the untreated and saline groups, and no obvious local ulcers or swelling were found, but the other two groups without stem cells transplanted showed a distinct gingival defect area. At 2 weeks, the height of the defect area was significantly increased in the stem cell transplantation group, whereas the two groups without stem cells showed a locally congested or grayish gingival defect area. Three weeks after transplantation, the morphology and color of the local gingival tissue of the cell transplanted group was similar to normal gingival tissue, and the gingival height was the same as that of the normal control group. However, gingival defects in the untreated and saline groups did not heal completely (Fig. 3a). Four weeks after transplantation, gingival tissues were stained by immunohistochemistry (Fig. 3b). The transplanted cells are observed as multiple masses, located below the gingival epithelium, and no similar cell mass can be seen in normal gingival tissue under hematoxylin–eosin (HE) staining. Transplanted cell masses under human Mito staining were positive, which showed that the transplanted human fetal GMSCs survived in the gingival tissues of rats. On rat CD20 and CD3 staining, transplanted cells masses were negative and no clearly visible positive cells were present around the transplanted cells, indicating that there was no immune cell infiltration after cell transplantation.

### Tumorigenicity assay of human fetal GMSCs

**Fig. 4:**
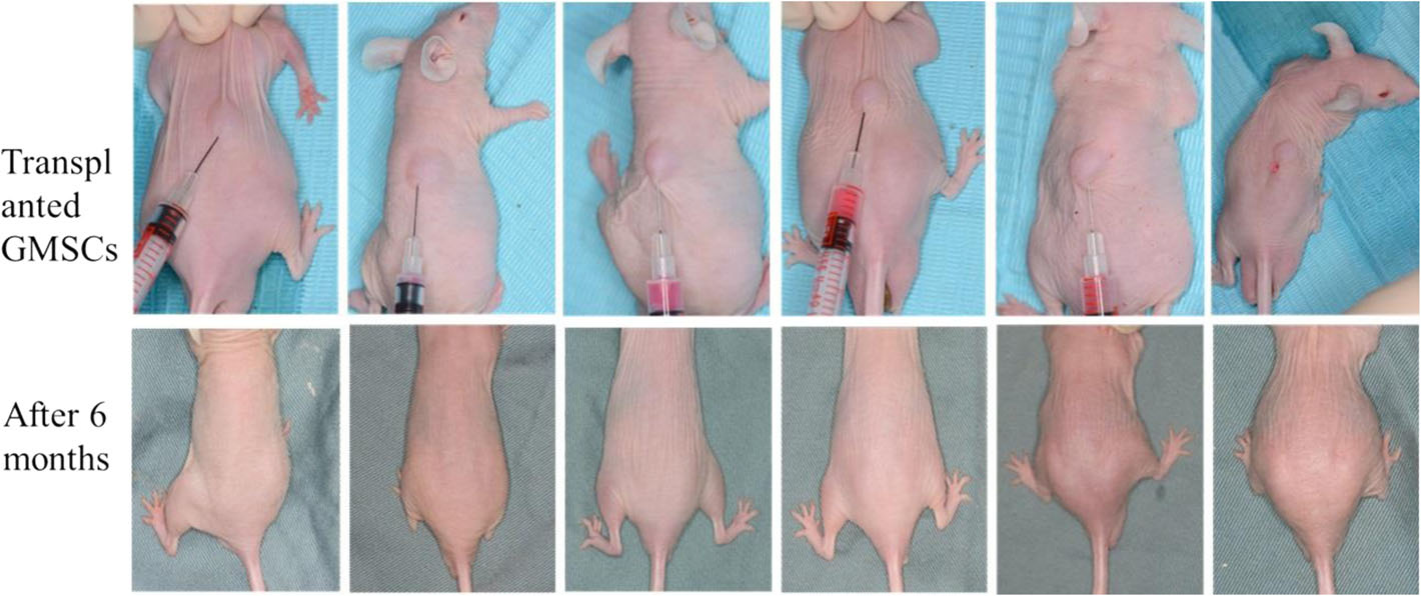
Tumorigenicity assay of human fetal GMSCs. Human fetal GMSCs subcutaneously inoculated into the backs of nude mice, and grafts observed every 3 days. Continuous observation for 6 months showed no abnormal mass in any of the mice. GMSC gingival mesenchymal stem cell

After inoculation of 2 × 10^6^ cells in the back, mice were observed every 3 days for 6 months. No tumors formed in any of the six mice (Fig. 4).

### Immunogenicity assay of human fetal GMSCs

**Fig. 5:**
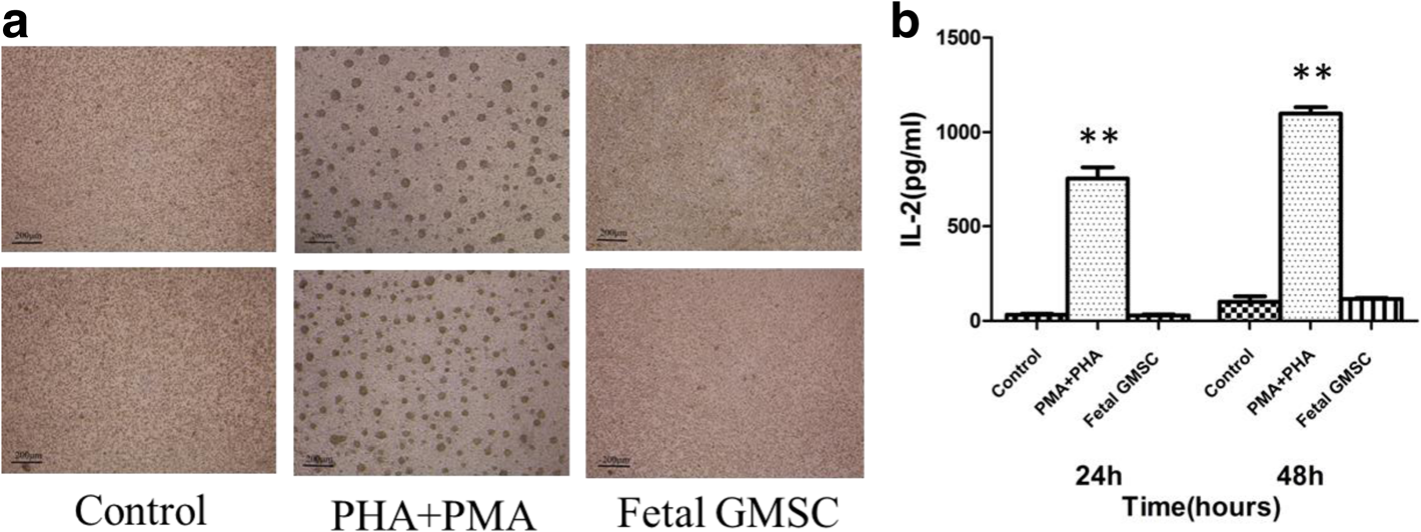
Immunogenicity assay of human fetal GMSCs. **a** Human fetal GMSC lysates cocultured with human PBMCs, PHA + PMA-induced PBMCs used as positive control. After coculture for 24 and 48 h, there was no cell aggregation in the control and stem cell lysate groups, but the PHA + PMA group showed mass cell aggregation. **b** After coculture with human PBMCs for 24 and 48 h, IL-2 was measured by ELISA. Data presented as mean ± SD from four independent experiments (**P < 0.01). GMSC gingival mesenchymal stem cell, IL interleukin, PHA phytohemagglutinin, PMA phorbol myristate acetate

In the mixed lymphocyte reaction, cell lysates of human fetal GMSCs were cocultured with human PBMCs, and after 24 and 48 h there was no cell aggregation in the control and stem cell lysate groups. However, the PHA + PMA group showed obvious cell aggregation. ELISA results after 24 h of coculture showed that IL-2 produced by the GMSC group was 29.04 pg/ml and that produced by the PHA + PMA group was 753.84 pg/ml, which was statistically significant (*P* < 0.01). IL-2 produced by the control group was 32.99 pg/ml, which was not significantly different from the GMSC group. After 48 h of coculture, IL-2 produced by the GMSC group was 117.22 pg/ml and that produced by the PHA + PMA group was 1099.22 pg/ml, which was statistically significant (*P* < 0.01). IL-2 produced by the control group was 101.66 pg/ml, which was not significantly different from the GMSC group. These results showed that human fetal GMSCs had low immunogenicity (Fig. 5).

## Discussion

Periodontal disease is a common and frequent inflammatory disease of the periodontal tissue, the destruction of which is a main cause of adult tooth loss. Currently there are no effective treatments for severe periodontal tissue defects. The use of tissue engineering to achieve true regeneration of periodontal tissue is the future direction of clinical treatment, but finding a convenient, reliable source of seed cells is an issue to overcome.

Periodontal ligament stem cells, gingival mesenchymal stem cells, stem cells from human exfoliated deciduous teeth, stem cells from apical papilla, and dental follicle stem cells have been used for periodontal regeneration in experimental animals, and different microenvironments can induce stem cells to form different tissues [3–8]. Autologous dental pulp stem cells have been combined with a collagen sponge scaffold and transplanted into the space of extracted teeth, regenerating autogenous bone and achieving good periodontal tissue repair [9]. Feng et al. [10] transplanted human autologous periodontal ligament progenitors mixed with bone-grafting material into deep periodontal pockets of patients. After 72 months, healthy periodontal tissue regeneration was found.

Nestin, Oct4, and NANOG are markers of embryonic stem cells and play an important role in the maintenance of pluripotency and self-renewal of cells [11]. In this study, 98.23% of human fetal GMSCs were Oct4-positive cells, 99.87% were nestin-positive cells, and 58.77% were NANOG-positive cells, indicating that the human fetal GMSCs had self-renewal potential and pluripotency characteristics. Vimentin was expressed in mesenchymal cells, and CD105 and CD90 are typical MSC-associated surface markers. In GMSCs, positive expression of vimentin (99.88%), CD105 (95.41%), and CD90 (98.65%) showed that these cells were mainly derived from mesenchymal stem cells.

In periodontal lesions with site specificity, the most frequently involved teeth are mandibular incisors and maxillary molars. With rat animal models of periodontal disease, most scholars choose relatively stable molars as experimental teeth. However, to facilitate observation, some researchers choose mandibular incisors [12]. In this study, we chose the mandibular incisor labial gingiva as the experimental site, since this area can be accessed easily to prepare a gingival defect model and for cell transplantation, and can be observed easily so that the animal does not require repeated anesthesia. Experimental periodontal tissue defects can be divided into acute defects and chronic defects. Acute defects are achieved by removal of periodontal tissue with surgery, while chronic defects are realized by applying continuous force and a local plaque retention environment [13]. The onset of chronic defects as simulated by the periodontal disease process is more advantageous for pathogenesis research. However, to study a new therapeutic method, we chose acute defects that had fewer confounding impacts. There is no ideal animal model for gingival defect to simulate human gingival recession. In this study, the animal model was established by surgical technique, which could simulate gingival recession very well but not bone loss.

Human fetal GMSCs were transplanted to the labial gingiva defects of the anterior teeth of rats. One week after transplantation the defect area clearly decreased, and after 3 weeks the morphology and color of local gingival tissue was normal. Four weeks after transplantation, human fetal GMSCs had effectively promoted the regeneration of gingival tissue, showing that stem cells were effective. Moreover, human mitochondrial staining was positive in the graft tissue, showing that the transplanted human fetal GMSCs survived in the gingival tissues of rats and may have been the main seed cell that promoted regeneration.

Beside efficacy, safety is another key factor for the clinical application of stem cells. Continuous observation for 6 months of human fetal GMSCs showed no tumorigenicity. Immunogenicity analysis of human fetal GMSCs showed that the cells did not cause allogeneic lymphocyte focal aggregation and did not lead to a large release of IL-2, suggesting low immunogenicity. Staining of CD3 and CD20 in transplanted tissue was negative, showing that cell transplantation did not induce local immune response.

At present, the subepithelial connective tissue graft is the main method for the treatment of human Miller class I or II single or multiple gingival recessions, but two surgical areas will occur in the mouth at the same time. Stem cell transplantation can successfully repair the gingival tissue without any trauma, and cell therapy is a reliable and successful treatment for human Miller class I or II single or multiple maxillary gingival recessions [14]. Stem cells derived from fetal gingival tissue have advantages over other types of cells. Human fetal tissue-derived stem cells are highly undifferentiated and have strong proliferation ability in vitro. Fetal gingival tissue-derived stem cells may contain more precursor cells for gingival differentiation. Therefore, these cells can differentiate into gingival cells automatically in vivo. In this study, we demonstrated that human fetal GMSCs can effectively regenerate gingival tissue, and histological results showed that the structure of new tissue was like that of normal gingiva.

Fetal stem cells are less derived than adult stem cells and have stronger capacity of proliferation and multilineage differentiation. Fetal stem cells also have an engraftment advantage over adult cells, and fetal recipients are more permissive to allogeneic donor grafts than adult recipients [15]. Due to the expression of MHC II after 14 gestational weeks [16, 17], stem cells derived from younger fetal tissue should have lower immunogenicity. In this study, human fetal GMSCs were derived from fetal tissue at 12 gestational weeks and showed very low immunogenicity; these may be ideal seed cells for periodontal regeneration.

Self-renewal and multilineage differentiation potential are the main characteristics of fetal tissue-derived stem cells. Fetal pancreatic tissue stem cells have achieved good experimental results in the treatment of diabetes mellitus [18, 19], and embryonic retinal stem cells in experimental treatments of retinitis pigmentosa can form normal retinal cells, including cones, rods, and light cells [20]. These results indicate that human fetal tissue-derived stem cells have promising clinical applications. At present, in studies of periodontal regeneration, adult gingival stem cells are mainly used as gingival mesenchymal stem cells. We obtained gingival mesenchymal stem cells from fetal gingival tissue, a source consistent with the mesenchymal stem cells derived from adults, but these are in a more primitive undifferentiated state and have stronger multilineage differentiation capacity. The use of fetal stem cells derived from gingival tissue has not been reported previously, so our results provide an experimental basis for the use of these cells in the treatment of gingival tissue defects. Human fetal stem cells may have more effects other than repair of the soft tissue. We will continue studying fetal stem cells in periodontal regeneration, including their effect on alveolar bone defects.

## Conclusion

Our results show that human fetal GMSCs are safe, of low immunogenicity, and can effectively promote the regeneration of periodontal tissue. Fetal stem cells may serve as a new kind of seed cell for the treatment of severe human gingival tissue defects.

## Abbreviations

bFGF: Basic fibroblast growth factor
DMEM: Dulbecco’s modified Eagle’s medium
EDTA: Ethylenediaminetetraacetic acid
EGF: Epidermal growth factor
ELISA: Enzyme-linked immunosorbent assay
GMSC: Gingival mesenchymal stem cell
IL-2: Interleukin-2
LIF: Leukemia inhibitor factor
Mito: Mitochondrial
PBMC: Peripheral blood mononuclear cells
PBS: Phosphate-buffered saline
PHA: Phytohemagglutinin
PMA: Phorbol myristate acetate
